# Diagnostic Accuracy of Clinical Diagnostic Scoring Systems for Childhood Tuberculosis: A Systematic Review and Meta-analysis

**DOI:** 10.1093/ofid/ofad624

**Published:** 2023-12-11

**Authors:** Michael Kakinda, Ronald Olum, Joseph Baruch Baluku, Felix Bongomin

**Affiliations:** Clinical Directorate, Joint Clinical Research Center, Kampala, Uganda; Department of Medicine, St Francis Hospital Nsambya, Kampala, Uganda; Network for Infectious Diseases Epidemiology and Research (NIDER) Platform, Kampala, Uganda; Network for Infectious Diseases Epidemiology and Research (NIDER) Platform, Kampala, Uganda; Division of Pulmonology, Kiruddu National Referral Hospital, Kampala, Uganda; Makerere University Lung Institute, College of Health Sciences, Makerere University, Kampala, Uganda; Network for Infectious Diseases Epidemiology and Research (NIDER) Platform, Kampala, Uganda; Department of Medical Microbiology and Immunology, Faculty of Medicine, Gulu University, Gulu, Uganda

**Keywords:** diagnostic scoring system, diagnostic test accuracy, pediatric TB

## Abstract

**Background:**

Diagnosis of childhood tuberculosis (TB) poses several challenges. Therefore, point-based scoring systems and diagnostic algorithms have been developed to improve the diagnostic yields in this population. However, there are no updated systematic reviews of the existing childhood TB scoring systems and algorithms. Hence, we systematically reviewed the diagnostic accuracy of the childhood TB diagnostic scoring systems and algorithms.

**Methods:**

We systematically searched PubMed, CINAHL, Embase, Scopus, and Google Scholar databases for relevant articles published until 30 March 2023. QUADAS-2 was used to assess their study quality. Diagnostic accuracy measures (ie, sensitivity, specificity, diagnostic odds ratio, positive and negative likelihood ratios) were pooled using a random-effects model.

**Results:**

We included 15 eligible studies, with a total of 7327 study participants aged <15 years, with 10 evaluations of childhood TB diagnostic scoring systems and algorithms. Among these algorithms and scoring systems, only 3 were evaluated more than once. These were the Keith Edwards scoring system with 5 studies (sensitivity, 81.9%; specificity, 81.2%), Kenneth Jones criteria with 3 studies (sensitivity, 80.1%; specificity, 45.7%), and the Ministry of Health–Brazil algorithm with 3 studies (sensitivity, 79.9%; specificity, 73.2%).

**Conclusions:**

We recommend using the Keith Edwards scoring system because of its high sensitivity and specificity. Further research is necessary to assess the effectiveness of scoring systems and algorithms in identifying TB in children with HIV and malnutrition.

Tuberculosis (TB) is an infectious disease of global public health significance that is caused by the *Mycobacterium tuberculosis* complex. It is one of the leading causes of illness and death worldwide [1, 2]. In 2021, an estimated 10.6 million people were estimated to have TB worldwide. Children and young adolescents aged <15 years represented an estimated 10.4% of all these cases, translating to >1.1 million children with TB [2]; however, only 448 000 (40.7%) were notified [1]. The remaining patients were undiagnosed, misdiagnosed, or not reported. Approximately 1 in 5 children (<15 years old) who developed TB lost their lives [1]. A modeling study estimated that 96.0% of TB deaths in children occur because they do not receive appropriate treatment [3].

TB diagnosis in children has been missed for several reasons. The symptoms of childhood TB are nonspecific and tend to overlap with those of other common childhood illnesses. These include pneumonia, human immunodeficiency virus (HIV)–associated lung diseases, and malnutrition [4]. Hence, for many children, the diagnosis of TB as a cause of illness is not considered. For those in whom it is considered, biological samples are often difficult to collect [5]. The available diagnostic tests, when available, have low diagnostic accuracy [6] because most children have too few bacilli to be detected [2].

The sensitivity of *M tuberculosis* culture, the reference standard for diagnosis, rarely exceeds 30%–40% [7, 8]. Therefore, confirming childhood TB is an exception and not the norm. Owing to the challenges in diagnosing childhood TB, individual clinical signs and symptoms, radiological studies, laboratory examinations, point-based scoring systems, and/or diagnostic algorithms have been developed to aid its diagnosis [9, 10].

The first diagnostic scoring system for childhood TB was developed in 1969 [11]; since then, there have been several models with various iterations. Their major objective was to provide a consistent and accurate way to diagnose childhood TB clinically, especially in resource-limited settings [9]. Although diagnostic scoring systems for childhood TB have been in use for >53 years, their validity and reliability remain unclear. Different diagnostic scoring systems have been used in different locations [12] and may not have been evaluated. There is also limited literature on the evaluation of diagnostic scoring systems among malnourished children and those coinfected with HIV and TB [9, 10].

The last review of diagnostic scoring systems for childhood TB called for a consistent inclusion criterion and for them to be evaluated in multiple geographical locations and patient populations [9]. The last review was conducted 10 years ago [9], and there is a likelihood of new literature over the said period. We conducted a systematic review of the diagnostic accuracy of childhood TB diagnostic scoring systems and algorithms.

## METHODS

### Protocol and Registration

We developed, conducted, and reported this review following the Preferred Reporting Items for Systematic Reviews and Meta-Analyses of Diagnostic Test Accuracy (PRISMA-DTA) [13]. The protocol was registered in PROSPERO (CRD42022367049) and published in a peer-reviewed journal [14].

### Eligibility Criteria

The eligibility criteria for studies were formulated based on the participants (population), index test, comparator test, and target condition (PICT) for the review question. We included studies that evaluated the diagnostic accuracy of childhood TB scoring systems in terms of their sensitivity and specificity against a reference standard and had sufficient information for the construction of a diagnostic 2 × 2 table (true positive, true negative, false positive, false negative). Given that childhood TB has an imperfect reference standard, we used a composite reference standard.

The participants were children aged <15 years with presumptive TB who underwent a diagnostic TB scoring test or TB diagnostic algorithm. Only original studies published in the English language until 30 March 2023 were included. These were peer-reviewed prospective or retrospective cohort, cross-sectional, interventional, and case-control studies that addressed the review questions. Editorials, letters to editors, and conference abstracts were excluded because they were unlikely to contain sufficient data. The target condition was intrathoracic childhood TB.

### Index Test

The index test was either a scoring system or diagnostic algorithm used to diagnose childhood TB. Where a diagnostic algorithm is a step-by-step method for making a diagnosis using a combination of symptoms, signs, or test results, whereas a scoring system is a numerical scale composed of signs, symptoms, and test results with an agreed cutoff to diagnose the disease. These often consider individual clinical signs and symptoms, radiological studies, and laboratory examinations, which are used to construct a point-based scoring system or diagnostic algorithm.

### Reference Standard (Comparator Test)

Any attempt to confirm a TB diagnosis was used as the reference standard. This was not limited to sputum culture, GeneXpert MTB/RIF or Ultra, TB lipoarabinomannan, and other tests that confirmed TB, as TB does not have an ideal reference standard. We also considered unconfirmed TB as defined by Graham et al [15], where bacteriological confirmation was not obtained but there were at least 2 of the following features: chest radiograph consistent with TB, immunological evidence of *M tuberculosis* infection, or positive response to anti-TB treatment. These tests were used as the composite reference standard for the diagnosis.

### Search Strategy

We conducted a systematic search for relevant articles using PubMed, Cumulative Index to Nursing and Allied Health Literature (CINAHL), Embase, Scopus, and Google Scholar databases. The search strategy included the following keywords: “paediatric tuberculosis,” OR “pediatric tuberculosis” OR “pediatric TB” OR “paediatric TB” OR “childhood tuberculosis,” OR “childhood TB” AND “diagnostic accuracy” OR “sensitivity” OR “specificity” AND “algorithm” OR “scor*” OR “clinical scor*” OR “diagnostic scor*,” OR “diagnostic screen*.” Furthermore, we conducted a freehand search for relevant articles in the reference section of the articles included in the review to avoid missing eligible studies. We included studies published up to 30 March 2023.

### Data Screening and Extraction

The search results were exported to Zotero software, a research tool for collecting, organizing, and managing research publications [16]. They were then transferred to Rayyan Artificial Intelligence Software for Systematic Reviews and Meta-analysis, which was used to screen the titles and abstracts [17]. Our review team, comprised of 3 members (F. B., R. O., and M. K.), independently reviewed all the studies from the search and additional resources, which they coded as “include,” “exclude,” or “maybe.” The fourth reviewer, J. B. B., who was blinded to the other reviewers’ decisions, thoroughly reviewed the disagreements between them and made the final decision by consensus.

All eligible studies were transferred to Zotero. Full-text articles were narrowed down and assessed for inclusion using PICT questions as the inclusion criteria. The screened studies were placed into appropriate subfolders created in Zotero based on the decision to include, exclude, or maybe.

Final data were extracted from the included studies using a spreadsheet (Microsoft Excel 365; Microsoft Corporation, Redmond, Washington). The following information was extracted: authors, year of publication, age distribution, study design, diagnostic score, reference standard, country, sample size, setting, and outcome measures (ie, sensitivity and specificity). In case of missing data, the authors of the study were contacted for additional information. Studies that did not respond to the query were excluded.

M. K. and F. B. independently extracted the data, whereas R. O. and J. B. B. checked the extracted data to ensure completeness and accuracy. Upon disagreements, all 4 reviewers reviewed the final extracted data, and disagreements were resolved through discussion of the inclusion and exclusion criteria via the majority decision.

### Risk of Bias

The Quality Assessment of Diagnostic Accuracy Studies (QUADAS)-2 tool was used to assess the methodological quality of all studies included in this systematic review [18]. The QUADAS-2 consists of 4 key domains: patient selection, index test, reference standard, and flow and timing. We assessed all domains for the risk of bias potential using different signaling questions and the first 3 domains for applicability concerns. Based on this, the risk of bias was judged as “low,” “high,” or “unclear.” A summary of the QUADAS-2 results for all included studies is presented in tabular and graphical form. Two reviewers (M. K. and F. B.) independently assessed the risk of bias and study quality. Disagreements between the authors were resolved through discussion and consensus with other authors.

### Statistical Analysis and Data Synthesis

We extracted data from the included studies to construct a standard 2 × 2 table to calculate the sensitivity and specificity. The data were then entered into MetaDTA [19]. MetaDTA is a freely available interactive online application in which meta-analyses DTA studies plot the summary receiver operating characteristic (ROC) curve, incorporate quality assessment results, and allow prompt sensitivity analyses. Using a random-effects model, we used MetaDTA to calculate the pooled sensitivity and specificity of studies that evaluated the same scoring system or algorithm. Positive likelihood ratio, negative likelihood ratio, and diagnostic odds ratio were also calculated using MetaDTA. Higgins’ inconsistency index (*I*^2^) was used to measure heterogeneity (*I*^2^*>*50% indicating substantial heterogeneity) [20, 21] using Stata 17 software (StataCorp, College Station, Texas). We also created forest plots for sensitivity and specificity, and a hierarchical summary ROC curve using MetaDTA.

The included studies were grouped based on their diagnostic scores. MetaDTA was used to calculate the pooled sensitivity, pooled specificity, and false-positivity rate of the subgroups, whereas Stata was used for forest plots and heterogeneity.

### Patient Consent Statement

This research was exempt from ethical approval, given that this was a systematic review that used published data.

## RESULTS

### Selection and Characteristics of the Included Studies

**Figure 1.**
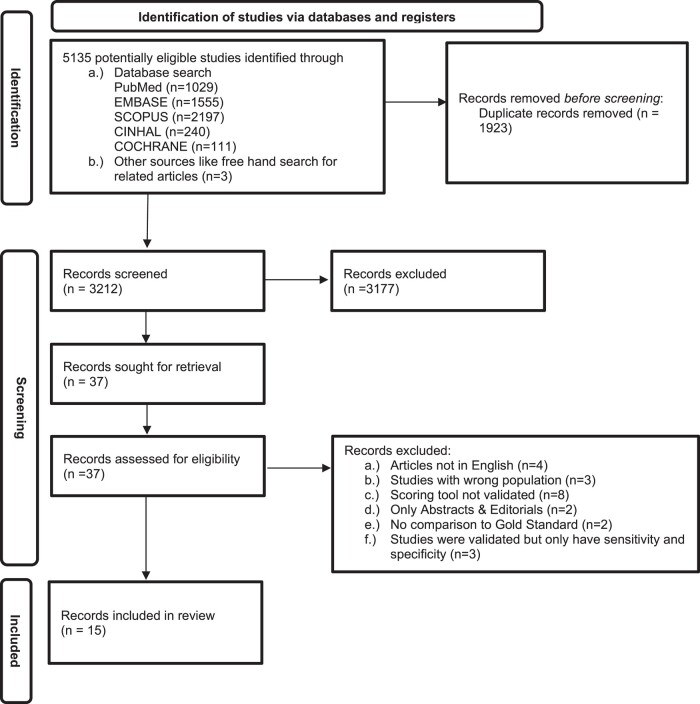
Preferred Reporting Items for Systematic Reviews and Meta-Analyses (PRISMA) 2020 flow diagram for diagnostic accuracy of childhood tuberculosis scoring systems and algorithms.

After performing a systematic search, we identified 5135 records. In total, 1923 duplicate records were removed. The rest (3212) underwent title and abstract screening. A total of 3177 records did not address the review question and were therefore excluded. Full-text screening was conducted on the remaining 37 records, of which 15 were included in the review. Of the 22 excluded records, 8 had a scoring system that was not evaluated, 4 articles were not in English, 3 studies were of the wrong population, 3 studies were evaluated but only reported a sensitivity and specificity, 2 studies were only abstracts and editorials, and in 2 studies, the scoring system was not compared to any reference standard (Figure 1).

Studies included in the diagnostic accuracy studies were conducted in Brazil (3), India (2), Pakistan (2), South Africa (1), Papua New Guinea (1), Uganda (1), Zambia (1), Tanzania (1), the Philippines (1), and 2 multicountry studies—1 with 4 countries (Burkina Faso, Cambodia, Cameroon, and Vietnam) and another with 12 countries (Bangladesh, Brazil, Burkina Faso, Cambodia, Cameroon, Mozambique, Kenya, Pakistan, Vietnam, Myanmar, Uganda, and South Africa), which enrolled 7327 study participants aged <15 years.

The Keith Edwards score was the most evaluated, with 5 studies among the included studies, followed by the Kenneth Jones criteria and Ministry of Health (MoH)–Brazil score, with 3 studies each. The rest had only 1 study each (International Union of Tuberculosis and Lung Disease [IUATLD] algorithm, MoH-Pakistan algorithm, Gunasekera algorithm, Ben Marais score, Tidjani score, Ghidey and Habte score, and Marcy algorithm). Most of the included studies evaluated only 1 scoring system, with 1 study comparing 2 scoring systems and another 4 (Table 1).

**Table 1.** Characteristics of Included Studies

Study	Year	Age Limit	Study Design	Sample Size	Country	Scoring System/Criteria	Reference Standard	HIV Status (Prevalence)
Cartaxo et al [22]	2013	<15 y	Cross-sectional	163	Brazil	MoH-Brazil (>30)	Radiology, TST, culture, microscopy	No testing
David et al [23]	2017	<15 y	Retrospective cohort	121	Brazil	Kenneth Jones, Tidjani, MoH-Brazil (>30), Ben Marais	CXR, contact, response to treatment, culture, SS	HIV^+^ (100%)
Farid et al [24]	2013	<15 y	Cross-sectional	100	Pakistan	Modified Kenneth Jones criteria	Smear microscopy, culture, biopsy, CXR, and CT	No testing
Gunasekera et al [25]	2021	<13 y	Retrospective cohort	478	South Africa	Gunasekera 2021 algorithm	Smear microscopy, GeneXpert, culture, and CXR	HIV^–^ (100%)
Gunasekera et al [26]	2023	<10 y	Individual meta-analysis	4178	Brazil, Vietnam, Pakistan, Bangladesh, Mozambique, Burkina Faso, Cambodia, Cameroon, Myanmar, Uganda, Kenya, Mozambique, South Africa	Gunasekera 2023 algorithm	Smear microscopy, GeneXpert, culture, CXR, and response to treatment	HIV^+^ (24%)
Kumar et al [27]	2013	<12 y	Cohort study	100	Pakistan	Kenneth Jones criteria, Pakistan-NTP algorithm	Response to treatment, CXR, SS	No testing
Lateo et al [28]	2003	<14 y	Cross-sectional	494	Philippines	IUATLD	Culture	No testing
Marcy et al [29]	2019	<13 y	Cohort study	438	Burkina Faso, Cambodia, Cameroon, Vietnam	Marcy algorithm	QFT, ultrasound, CXR, culture	HIV^+^ (100%)
Migliori et al [30]	1992	<5 y	Cross-sectional	210	Uganda	Ghidey and Habte	Smear microscopy, TST, CXR, contact, SS	No testing
Mutabazi et al [31]	2009	<15 y	Cross-sectional	252	Tanzania	Keith Edwards	Culture, GeneXpert	HIV^+^ (100%)
Narayan et al [32]	2003	<12 y	Cross-sectional	101	India	Keith Edwards	Culture, histology, CXR	HIV^+^ children excluded
Sant’Anna et al [33]	2006	<12 y	Case-control	141	Brazil	MoH-Brazil (>30)	Culture	No HIV testing
Sarkar et al [34]	2009	<12 y	Case-control	103	India	Keith Edwards	Smear microscopy, biopsy, CXR	No HIV testing
van Beekhuizen et al [35]	1998	<15 y	Cross-sectional	301	Papua New Guinea	Keith Edwards	CXR, TST, biopsy	No HIV testing
van Rheenen et al [36]	2002	<12 y	Cohort study	147	Zambia	Keith Edwards	Culture, smear microscopy, CXR, TST	HIV^+^ (100%)

Abbreviations: CT, computed tomography; CXR, chest radiograph; HIV, human immunodeficiency virus; IUATLD, International Union Against Tuberculosis and Lung Diseases; MoH, Ministry of Health; NTP, National Tuberculosis Program; QFT, QuantiFERON-TB Gold; SS, signs and symptoms; TST, tuberculin skin test;.

### Methodological Quality of Studies

#### Patient Selection

Of the 15 studies assessed in the patient selection domain, 9 (60%) were rated as having a low risk of bias, while 5 (33.3%) and 1 (0.1%) were found to have a high risk and unknown risk of bias, respectively. In studies with a low risk of bias, patients were randomly selected, eliminating this bias. Three of the 5 (60%) studies deemed high risk in this domain were case-control studies [22, 32, 33] while the remaining 2 studies excluded all patients with any cause of immunosuppression, which is a risk factor for TB [27, 31].

#### Index Test

Conduct or interpretation of an index test was considered low risk in 12 of the 15 (80.0%) assessed studies. The remaining 3 studies had an unknown risk of bias. Studies with a low risk in the index test domain were interpreted without knowledge of the reference standard, whereas insufficient information was available to determine the risk of bias for studies with an unknown risk. All studies included in the review, index test, and conduct of interpretation matched the review questions.

#### Reference Standard

All studies evaluated under the reference standard domain had a high risk of bias. Most index tests had at least 1 component of the reference standard. Therefore, it is unlikely that the target condition and its interpretation were made without prior knowledge of index test results.

#### Flow and Timing

An evaluation was carried out based on the presence of an appropriate time gap between the index test and the reference standard, and whether all patients received the same reference standard and were included in the analysis. Ideally, the reference standard should be used immediately after the index test. However, this does not apply to pediatric TB, where a part of the reference standard may be a response to treatment. This makes the ideal time between the index test and the reference standard at least 2 months after initiation of treatment.

**Figure 2.**
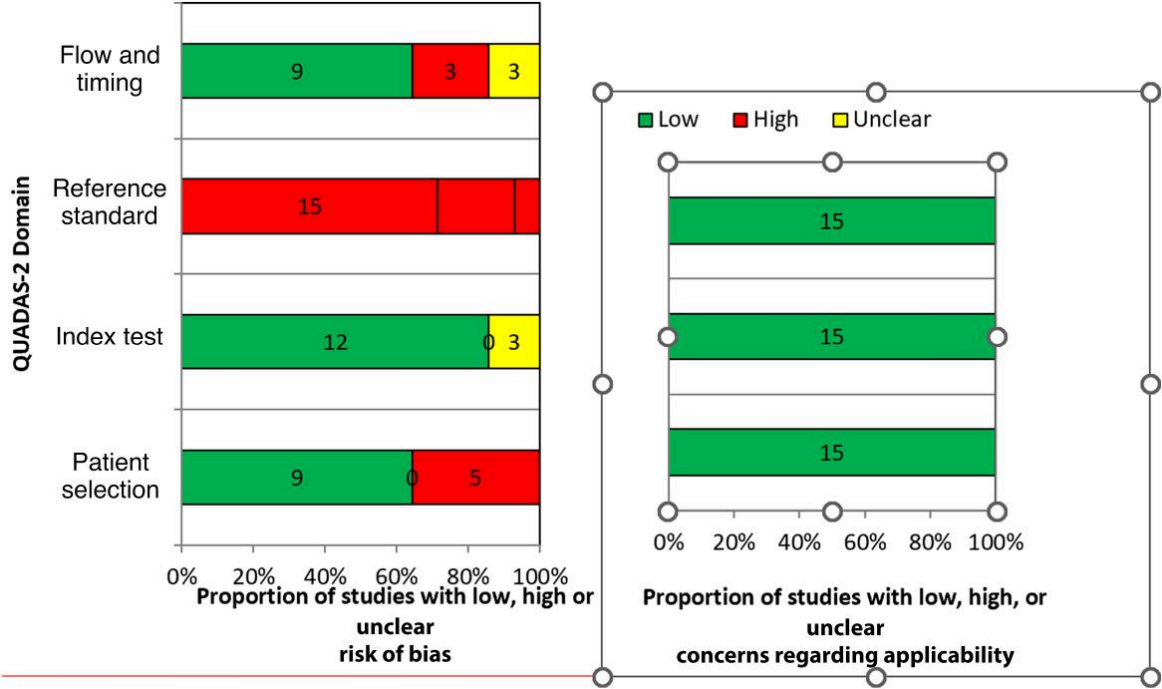
Graphical representation of the methodological quality of the included studies.

Ninety percent of the 15 studies were found to have low risk in the flow and timing domains. These patients were observed to have an appropriate interval between the index test and the reference standard, with all patients utilizing the same reference standard, and were included in the analysis. However, 3 studies were determined to be at high risk in the flow and timing domains [22, 23, 34]. They were identified as having an inappropriate interval between the index test and the reference standard. Additionally, van Beekhuizen et al did not use the same reference standard for all the patients included in the study [35]. The remaining studies had unclear risk in this domain. In the case of Gunasekera et al [25, 26], it was unlikely that all the studies used the same reference standard, while for Kumar et al [27], there was an inappropriate interval between the index test and reference standard because the response to treatment was part of the reference standard and they did not offer a time period considered [25, 26]. The methodological quality of the included studies is presented in Table 2 and Figure 2.

**Table 2.** Summary of Risk of Bias and Applicability Concerns for Each Included Study in Each Separate Domain

Study	Risk of Bias				Applicability Concerns		
	Patient Selection	Index Test	Reference Standard	Flow and Timing	Patient Selection	Index Test	Reference Standard
Cartaxo et al [22]	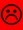	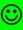	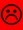	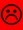	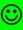	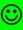	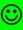
David et al [23]	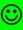	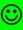	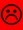	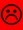	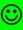	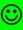	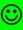
Farid et al [24]	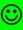	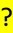	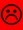	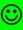	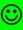	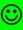	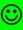
Gunasekera et al [25]	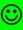	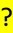	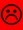	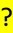	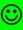	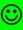	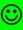
Gunasekera et al [26]	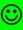	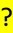	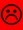	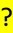	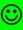	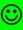	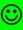
Kumar et al [27]	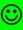	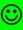	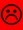	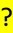	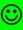	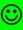	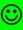
Lateo et al [28]	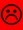	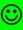	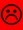	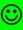	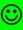	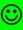	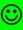
Marcy et al [29]	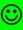	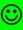	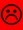	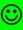	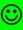	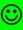	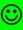
Migliori et al [30]	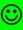	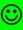	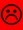	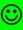	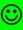	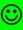	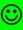
Mutabazi et al [31]	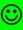	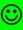	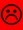	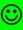	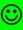	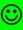	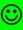
Narayan et al [32]	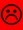	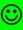	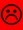	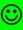	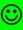	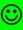	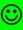
Sant’Anna et al [33]	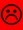	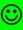	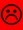	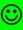	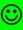	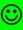	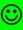
Sarkar et al [34]	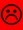	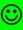	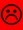	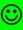	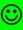	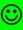	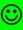
van Beekhuizen et al [35]	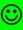	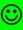	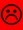	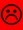	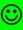	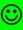	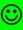
van Rheenan et al [36]	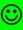	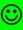	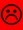	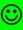	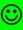	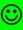	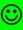

Key: 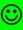, low risk; 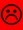, high risk; 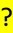, unclear risk.

#### Diagnostic Accuracy of Clinical Diagnostic Scoring Systems

Table 3 summarizes the true positive, false positive, true negative, and false negative rates; sensitivity and specificity; positive and negative likelihood ratios; and diagnostic odds ratio of each clinical diagnostic scoring system included in the systematic review.

**Table 3.** Summary of Diagnostic Accuracy Data for the Included Studies

Study	Year	TP	FN	FP	TN	Total No. of Events	Sensitivity	Specificity	Positive LR	Negative LR	DOR
Cartaxo et al [22]	2013	44	12	33	74	163	78.60%	69.20%	2.6	0.31	8.25
David et al [23] (KJC)	2017	43	21	9	9	82	67.20%	50.00%	1.3	0.66	2.05
David et al [23] (Tidjani)	2017	53	11	4	4	72	82.80%	50.00%	1.7	0.34	4.81
David et al [23] (MoH-Brazil)	2017	51	9	31	31	122	85.00%	50.00%	1.7	0.30	5.67
David et al [23] (BM)	2017	60	4	40	40	144	93.80%	50.00%	1.9	0.12	15.13
Farid et al [24]	2013	17	6	43	34	100	73.90%	44.20%	1.3	0.59	2.24
Gunasekera et al [25]	2021	219	23	156	80	478	90.50%	33.90%	1.4	0.28	4.89
Gunasekera et al [26]	2023	1521	290	2035	872	4178	83.99%	30.00%	1.20	0.53	2.25
Kumar et al [27] (KJC)	2013	84	8	4	4	100	91.30%	50.00%	1.8	0.17	10.49
Kumar et al [27] (Pakistan-NTP)	2013	82	4	9	5	100	95.30%	35.70%	1.5	0.13	11.26
Lateo et al [28]	2003	38	6	137	313	494	86.40%	69.60%	2.8	0.20	14.54
Marcy et al [29]	2019	178	23	52	134	387	88.60%	72.00%	3.2	0.16	19.98
Migliori et al [30]	1992	30	14	1	165	210	68.20%	99.40%	113.7	0.32	355.30
Mutabazi et al [31]	2019	10	3	24	215	252	76.90%	90.00%	7.7	0.26	29.96
Naryan et al [32]	2003	59	6	4	32	101	90.80%	88.90%	8.2	0.10	79.05
Sant’Anna et al [33]	2006	40	13	5	83	141	75.50%	94.30%	13.2	0.26	50.98
Sarkar et al [34]	2009	45	8	11	39	103	84.90%	78.00%	3.9	0.19	19.93
van Beekhuizen et al [35]	1998	68	41	9	169	287	62.40%	94.90%	12.2	0.40	30.88
Van Rheenen et al [36]	2002	66	9	54	18	147	88.00%	25.00%	1.2	0.48	2.44

Abbreviations: BM, Ben Marais; DOR, diagnostic odds ratio; FN, false negative; FP, false positive; KJC, Kenneth Jones Criteria; LR, likelihood ratio; MoH, Ministry of Health; NTP, National Tuberculosis Program; TN, true negative; TP, true positive.

**Figure 3.**
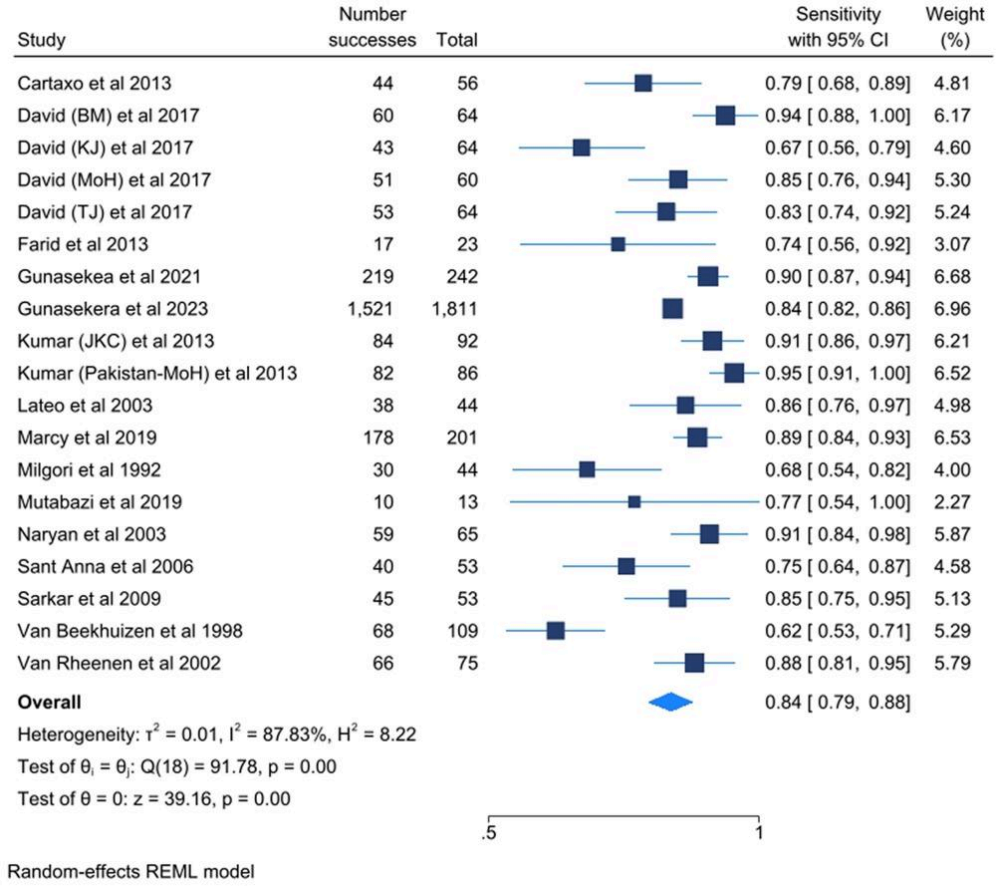
Forest plots for the sensitivity of the selected studies and their heterogeneity. Abbreviations: CI, confidence interval; KJC, Kenneth Jones Criteria; MoH, Ministry of Health; REML, Random Effect Model; TJ, Tidjan Scoring System.

**Figure 4.**
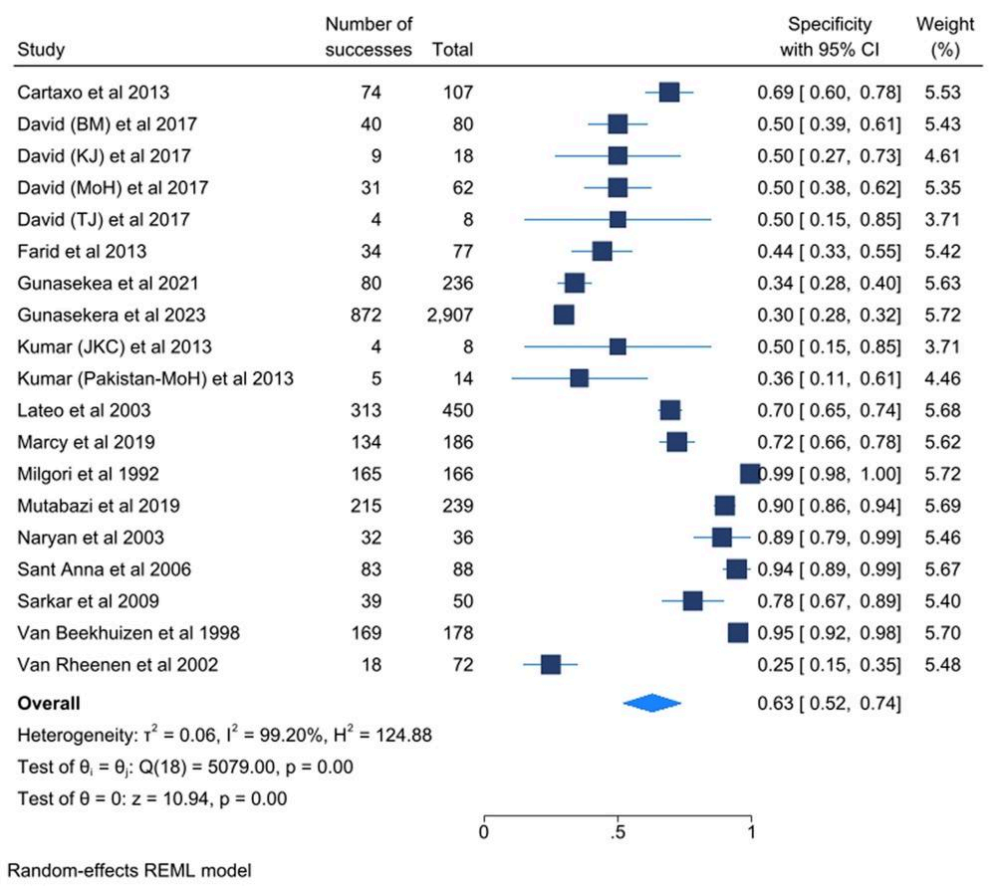
Forest plots for the specificity of the selected studies and their heterogeneity. Abbreviations: BM, Ben Marais Scoring System; CI, confidence interval; JK, Kenneth Jones Criteria; MoH, Ministry of Health; REML, Random Effects Model; TJ, Tidjani Scoring System.

**Figure 5.**
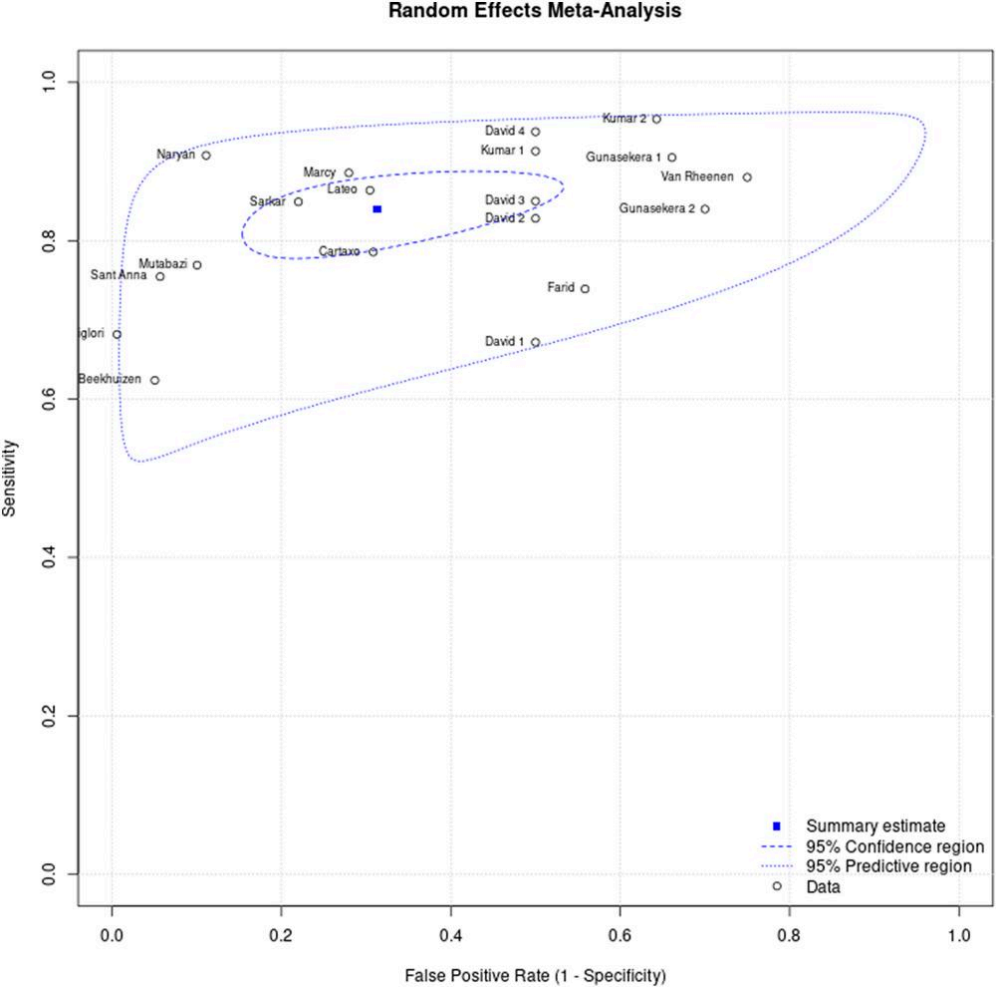
The hierarchical summary receiver operating characteristic curve for clinical diagnostic scoring systems for childhood tuberculosis. Where: David 1 (Kenneth Jones Criteria), David 2 (Tidjani), David 3 (MoH-Brazil), David 4 (Ben Marais), Gunasekera 1 (Gunasekera Algorithm 2021), Gunasekera 2 (Gunasekera Algorithm 2023), Kumar 1 (Kenneth Jones Criteria), Kumar 2 (MoH-Pakistan).

Figures 3 and 4 show the sensitivity and specificity of the included studies and their 95% confidence intervals (CIs), respectively. Figure 5 shows a summary of the clinical diagnostic scoring systems and algorithms for childhood TB their Test accuracy (sensitivity and false positivity rate), 95% CI, and 95% predictive region.

#### Subgroup Analysis

Table 4 summarizes the pooled sensitivity and specificity of the clinical diagnostic scoring systems for childhood TB subgroup analysis.

**Table 4.** Pooled Sensitivity and Specificity for Clinical Diagnostic Scoring Systems and Algorithms for Childhood Tuberculosis

Scoring System/Algorithm	No. of Studies Analyzed	Sensitivity					Specificity				
		Events	Total	Pooled Sensitivity (95% CI)	Heterogeneity		Events	Total	Pooled Specificity (95% CI)	Heterogeneity	
					*I* ^2^	*P* Value				*I* ^2^	*P* Value
Ben Marais	1	60	64	93.8% (84.8%–98.3%)	NA	NA	40	80	50.0% (39.0%–61.0%)	NA	NA
Ghidey and Habte	1	30	44	68.2% (52.4%–81.4%)	NA	NA	165	166	99.4% (99.4%–98.2%)	NA	NA
Gunasekera (2021)	1	219	242	90.5% (86.1%–93.9%)	NA	NA	80	236	33.9% (27.9%–39.9%)	NA	NA
Gunasekera (2023)	1	1521	1811	83.9% (82.1%–86.3%)	NA	NA	872	2907	30.0% (28.0%–32.0%)	NA	NA
IUATLD	1	38	44	86.4% (72.7%–94.8%)	NA	NA	313	450	69.6% (65.3%–73.8%)	NA	NA
Keith Edwards	5	248	315	81.9% (70.7%–89.5%)	84.70%	.001	473	575	75.5% (50.4%–100%)	98.90%	<.001
Kenneth Jones criteria	3	144	179	80.1% (63.1%–90.3%)	83.10%	.001	47	103	45.6% (36.0%–55.2%)	0	.875
Marcy algorithm	1	178	201	88.6% (83.3%–92.6%)	NA	NA	134	186	72.0% (65.6%–78.5%)	NA	NA
MoH-Brazil	3	135	169	79.9% (71.7%–86.2%)	0.00%	.406	188	257	71.7% (46.5%–96.8%)	96.4%	<.001
Pakistan-NTP	1	82	86	95.4% (88.5%–98.7%)	NA	NA	5	14	35.7% (10.6%–60.8%)	NA	NA
Tidjani	1	53	64	82.8% (71.3%–91.0%)	NA	NA	4	8	50.0% (15.4%–84.6%)	NA	NA

Abbreviations: CI, confidence interval; IUATLD, International Union Against Tuberculosis and Lung Diseases; MoH, Ministry of Health; NA, Not Applicable; NTP, National Tuberculosis Program.

Ten diagnostic scoring systems were considered for the subgroup analysis. These were the Keith Edwards scoring system (5), Kenneth Jones criteria (3), MoH-Brazil algorithm (3), Pakistan–National TB Program algorithm (1), Ben Marais criteria (1), Gunasekera 2021 algorithm (1), Gunasekera 2023 algorithm (1), Marcy algorithm (1), IUATLD algorithm (1), Tidjani scoring system (1), and the Ghidey and Habte scoring system (1). The range of sensitivities for the scoring systems was 68.18%–93.35% while that of specificities was 35.71%–99.40%. The Keith Edwards scoring system had a 5-score system included in the systematic review with a pooled sensitivity of 81.9% (95% CI, 70.7%–89.5%; *I*^2^ = 85.73%; *P* < .001) and a pooled specificity of 81.2% (95% CI, 55.30%–93.80%; *I*^2^ = 98.92%; *P* < .001). The other scores or algorithms that had multiple studies (3 each) included in the systematic review had a pooled sensitivity of 80.1% (95% CI, 63.4%–90.3%; *I*^2^ = 83.14%; *P* < .001) and 79.9% (95% CI, 71.7%–86.2%; *I*^2^ = 0.00%; *P* < .406) for the Kenneth Jones criteria and MoH-Brazil scoring system, respectively. The pooled specificity was 45.70% (95% CI, 35.6%–56.1%; *I*^2^ = 0.00; *P* < .875) for Kenneth Jones criteria and 73.2% (95% CI, 67.3%–78.5%; *I*^2^ = 96.42; *P* < .001) for the MoH-Brazil algorithm (Table 4).

## DISCUSSION

We set out to conduct a systematic review of the diagnostic test accuracy of the childhood TB diagnostic scoring systems and algorithms. This review included 15 studies, involving a total of 7327 participants aged <15 years, with 10 childhood TB diagnostic scoring systems and algorithms. Of the 10 scoring systems and algorithms, only 3 were evaluated more than once. These were the Keith Edwards algorithm (5), with a pooled sensitivity of 81.9% and a pooled specificity of 81.2%; Kenneth Jones criteria (3), with a pooled sensitivity of 80.1% and a pooled specificity of 45.7%; and the MoH-Brazil algorithm (3), with a pooled sensitivity of 79.9% and a pooled specificity of 73.2%. The Gunasekera 2023 algorithm had the largest sample size (4178) with a sensitivity of 83.9% and specificity of 30.0%.

The individual meta-analysis conducted by Gunasekera et al (2023) evaluated the performance of the Keith Edwards and the MoH-Brazil scoring systems in >4718 patients [26]. The study reported a sensitivity of 93% (95% CI, 80%–98%) and 90% (95% CI, 73%–93%) for the Keith Edwards scoring system and MoH-Brazil scoring system, respectively, which is comparable to our findings. However, the specificities for both of these scores were lower at 24% (95% CI, 9%–49%) for the Keith Edwards scoring system and 34% (95% CI, 14%–62%) for the MoH-Brazil scoring system. We speculate that the difference in specificity may be attributed to the high number of participants with nonspecific childhood illnesses, such as bacterial pneumonia, HIV, and malnutrition, which are similar to TB.

Ideally, a diagnostic test should differentiate between those with and without a target condition. However, no diagnostic test is perfect, so when adopting a diagnostic test, there is often a trade-off between patients who do not have the target condition but are incorrectly identified as positive (false positive) and those who have the target condition but are incorrectly identified as negative (false negative) [37, 38]. If we consider a population of 1000 children with a prevalence of 10% for TB and use scoring systems or algorithms that have been evaluated multiple times, the Keith Edwards scoring system results in 18 false negatives and 169 false positives, compared to 20 false negatives and 489 false positives from the Kenneth Jones criteria. The MoH-Brazil algorithm will have 20 false negatives and 241 false positives, while the Gunasekera 2023 algorithm [26], recommended by the World Health Organization [39], will have 16 false negatives and 630 false positives (Table 5). Given this trade-off between the probable false negatives and false positives, we recommend the use of the Kenneth Edwards scoring system for the clinical diagnosis of TB in primary healthcare facilities.

**Table 5.** Summary of Findings

Diagnostic Accuracy of Clinical Diagnostic Scoring Systems for Childhood Tuberculosis	
Patients/population	Children <15 y presumed to have TB
Prior testing	History (cough, fever)
Setting	Mostly inpatient and outpatient
Index test	Diagnostic scoring system or algorithm
Importance	There is increased access to childhood TB diagnostics, especially in lower-level health facilities
Reference standard	Sputum culture, GeneXpert MTB/RIF, or Ultra & TB LAM with TB detected AND at least 2 of the following features: CXR consistent with TB OR immunologic evidence of TB infection OR positive response to anti-TB treatment
Target condition	Intrathoracic TB
Studies	Cohort studies, cross-sectional studies, interventional studies, and case-control studies that addressed the review question

	Summary Accuracy (95% CI)	No. of Participant (No. of Studies)	Median Proportion With Target Condition (IQR)	Implications Assuming 1000 Children			
				Prevalence			
				2.5%	5%	10%	20%
Keith Edwards	Sensitivity, 81.9% (70.7%–88.0%)	890 (5)	42.0% (5.2%–64.4%)	FN: 5 FP: 183	FN: 9 FP: 179	FN: 18 FP: 169	FN: 36 FP: 150
	Specificity, 81.2% (55.3%–93.8%)						
Kenneth Jones criteria	Sensitivity, 80.1% (63.4%–90.3%)	282 (3)	64.3% (23.0%–92.0%)	FN: 5 FP: 529	FN: 10 FP: 516	FN: 20 FP: 489	FN: 40 FP: 434
	Specificity, 45.7% (35.6%–56.1%)						
MoH-Brazil	Sensitivity, 79.9% (73.4%–85.6%)	436 (3)	40.4% (34.4%–49.2%)	FN: 5 FP: 261	FN: 10 FP: 255	FN: 20 FP: 241	FN: 40 FP: 214
	Specificity, 73.2% (67.3%–78.5%)						
Gunasekera algorithm (2023)	Sensitivity, 83.9% (82.1%–86.2%)	4178 (1)	43.34% (41.8%–44.9%)	FN: 4 FP: 682	FN: 8 FP: 665	FN: 16 FP: 630	FN: 32 FP: 560
	Specificity, 30.0% (28.0%–32.0%)						

Abbreviations: CI, confidence interval; CXR, chest radiograph; FN, false negative; FP, false positive; IQR, interquartile range; LAM, lipoarabinomannan; MoH, Ministry of Health; TB, tuberculosis.

The use of scoring systems and algorithms has been demonstrated to be beneficial in the diagnosis of childhood TB, particularly in lower healthcare facilities [40]. However, it is noteworthy that the diagnosis of childhood TB largely relies on the expertise and knowledge of the clinician, which may be limited to these facilities [41, 42]. Consequently, an ideal scoring system or algorithm should not only possess high sensitivity and specificity but also incorporate fewer diagnostics that may not be easily accessible at these facilities. The Keith Edwards scoring system primarily employs signs and symptoms, making it suitable for use at lower healthcare facilities where even a chest radiograph may not be available.

Further research is necessary to assess the effectiveness of scoring systems and algorithms in identifying TB in children with HIV, bacterial pneumonia, and malnutrition. These subpopulations may impact the accuracy of the diagnosis, as they present with symptoms similar to those of TB. Therefore, it is essential to rule out all potential differential diagnoses of childhood TB. Additionally, if a confirmatory test with high specificity is available, it should be conducted prior to using the scoring system or algorithm. This will help reduce false positives and, hence, improve the specificity.

This study had several limitations. Most of the scoring systems or algorithms included used chest radiography (except the Keith Edwards scoring system and Gunasekera 2023 algorithm) and tuberculin skin test (except the Tidjani scoring system and IUALTD), and all included bacteriological confirmation. These did bias their diagnostic accuracy because the reference standard also used the same tests as those elaborated by Graham et al. This study could also have missed some relevant studies.

In summary, TB is often diagnosed clinically in children. However, clinical diagnosis is dependent on the experience and knowledge base of the clinician [9]. This, together with diagnostics, is often lacking in lower healthcare facilities where children with TB are most likely to present first. We recommend the Keith Edwards scoring system because it has high sensitivity and specificity and can easily be used at primary care or lower health facilities.

## Supplementary Material

ofad624_Supplementary_Data

## Supplementary Data

Supplementary materials are available at *Open Forum Infectious Diseases* online. Consisting of data provided by the authors to benefit the reader, the posted materials are not copyedited and are the sole responsibility of the authors, so questions or comments should be addressed to the corresponding author.

## Notes

***Acknowledgments.*** We would like to thank Bethan Morgan of the Manchester University National Health Service Foundation Trust, who helped us search the relevant databases for the literature and helped start the process of writing this systematic review and meta-analysis.

***Financial support*.** F. B. is a Harvard University, Boston University, Northwestern University and University of New Mexico (HBNU) consortium NIH Fogarty Global Health Research Fellow, and his work is supported by the Fogarty International Center of the NIH (award number D43 TW010543).
