# Supplementary material for: Diagnostic Accuracy of Clinical Diagnostic Scoring Systems for Childhood Tuberculosis: A Systematic Review and Meta-analysis
Source: Open Forum Infect Dis. 2023 Dec 11;11(1):ofad624. doi: 10.1093/ofid/ofad624 (PMC10787364; doi:10.1093/ofid/ofad624)

**Appendix 1: Scoring systems and Algorithms**

**KEITH EDWARDS SCORING SYSTEM**

| **Feature** | **Score** | | |
| --- | --- | --- | --- |
|  | 0 | 1 | 2 |
| **Duration of illness (weeks)** | < 2 | 2-4 | >4 |
| **Nutrition (% of weight for age)** | >80 | 60-80 | <60 |
| **Family history of tuberculosis** | None | Reported by Family | Proven Sputum Positive |
| **Score for other features if present** | | | |
| **Feature** | | | **Score** |
| **Unexplained fever, night sweats, no response to malaria treatment** | | | 1 |
|
| Positive tuberculin test | | | 3 |
| **Lymph nodes: large, painless, firm, soft sinus in the neck/axilla** | | | 3 |
|
| Malnutrition, not improving after 4 weeks | | | 3 |
| **Central nervous system: change in temperament, fits with or without abnormal cerebrospinal fluid findings** | | | 3 |
|
| Joint Swellings, bone swellings, sinuses | | | 3 |
| **Unexplained abdominal mass, ascites** | | | 3 |
| **Angle deformity of spine** | | | 4 |

*A score >7 is indicative of TB.

**KENNETH JONES SCORING SYSTEM**

| **Score** | **+3** | **+2** | **+1** | **-1** |
| --- | --- | --- | --- | --- |
|  | Bacteriological confirmation | Radiological findings suggestive of TB | Nonspecific radiological findings | BCG vaccine in last 2 years |
|  | Tuberculosis granuloma | Signs and symptoms suggestive of TB | Signs and symptoms compatible with TB |  |
|  | TST ≥ 10 mm | TST 5-9 mm | Nonspecific granuloma |  |
|  |  | TST conversion – ≥10 mm response to 2^nd^ test | History of TB contact |  |
|  |  | History of bacteriologically confirmed TB contact | Child less than 2 years old |  |

* TST = tuberculin skin test

* A score of ≥7 indicates unquestionable TB, 5-6 points probable TB, 3-4 points possible TB, and 1-2 points not likely TB

**TIDJANI SCORING SYSTEM**

| Characteristic | Points |
| --- | --- |
| History of chronic cough | +1 |
| Meningeal signs | +1 |
| Matted peripheral lymph nodes | +3 |
| Hilar adenitis on X-ray | +3 |
| Infiltration with cavity | +4 |
| Miliary appearance | +4 |
| Erosion of one of more vertebrae | +3 |
| Positive culture | +7 |
| Rapid response to broad spectrum antibiotics | -6 |
| Radiological improvement without anti-tuberculosis treatment | -3 |
| Radiological improvement with anti-tuberculosis treatment | +3 |

* TB diagnosis is given for scores ≥6

**BRAZILIAN MoH SCORING SYSTEM**

| Characteristic | Points |
| --- | --- |
| Clinical manifestations  Fever or cough, lost energy, sputum, weight loss, or night sweats for >2 weeks  No symptoms or symptoms for <2 weeks  Respiratory infection improving with or without antibiotic treatment for common bacteria | +15  +0  -10 |
| Thoracic x-ray  Hilar adenomegaly or miliary pattern  Exudate or patch shadow (with or without cavitation) unaltered/worsening after > 2 weeks with antibiotic treatment for common bacteria  Exudate or patch shadow (with or without cavitation) <2 weeks  Normal | +15  +15  +5  -5 |
| Adult TB contact  Regular contact  None or occasional contact | +10  0 |
| BCG vaccination and TST  BCG ≥2 years ago or no BCG, TST >5 mm  BCG <2 years ago, TST >10 mm  TST ≤5 mm, regardless of BCG | +15  +15  0 |
| Nutritional status  Severe malnutrition (grade III)  Eutrophic or non-severe malnutrition | +5  0 |

* A score of 40 or more is a very likely case of TB, 30 or 35 indicates a possible TB case, and 25 or less is considered unlikely to be a TB case

**BEN MARAIS (BM) SYSTEM FOR CHILDHOOD TB DIAGNOSIS**

| Characteristic | Result |
| --- | --- |
| Risk factors for tuberculosis  Contact with person having confirmed, probable, or suspected tuberculosis  HIV Infection  Malnutrition (height and weight under 3^rd^ percentile)  Less than 5 years old and living in endemic area  Previous tuberculosis diagnosis  Pneumonia without improvement after 7-10 days of antibiotic treatment | Yes/No  Yes/No  Yes/No  Yes/No  Yes/No  Yes/No |
| Clinical criteria: Symptoms  Cough throughout the day, persisting for more than 21 days  Reported lack of weight gain or weight loss for three months or more  Fever, ≥38° C, and/or night sweats almost daily for more than 14 days | Yes/No  Yes/No  Yes/No |
| Clinical criteria: Physical symptoms  Lack of weight gain or weight loss for three months or more  Painless lymphadenopathy, with or without fistula  Distended abdomen with ascites, with or without hepatosplenomegaly  Signs of subacute meningitis with or without intracranial hypertension, movement disorder, focal deficit, altered mental state, seizure, and/or CSF with mononuclear pleocytosis, hyperproteinorrachia, hypoglycemia  Swollen joints, usually hip or knee, without pain  Kyphosis | Yes/No  Yes/No  Yes/No  Yes/No  Yes/No  Yes/No |
| Imaging criteria: Radiography and/or tomography  Ghon focus, pleural effusion, atelectasis, primary complex, consolidation, cavitary lesions, lymphadenopathy (hilar, paratracheal), and/or generalized lesions with and without miliary pattern | Yes/No |
| Immunological criteria: Tuberculin skin test  BCG ≥2 years ago or no BCG, TST >5 mm  OR  BCG <2 years ago, TST >10 mm | Yes/No |
| Microbiological Evaluation  Bacteriological confirmation by smear, culture, and/or PCR | Yes/No |

*Active TB confirmed: Positive microbiological evaluation

*Active TB probable: 3 out of 4 positive criteria

*Active TB highly possible: 2 out of 4 positive criteria, positive for ≥1 risk factor(s)

*Active TB possible: 1 out of 4 positive criteria, positive for ≥1 risk factor(s)

*Latent TB: TST positive without any other positive criteria

**PAKISTAN MoH SCORING SYSTEM**

| Condition | Scores |  |  |  |  |
| --- | --- | --- | --- | --- | --- |
|  | 1 | 2 | 3 | 4 | 5 |
| **Age** | < 5years |  |  |  |  |
| Close Contact^a^ | TB Suggestive | Clinically Positive | Bacteriological Positive |  |  |
| PEM/SAM^b^ | Yes | Not responding to Nurtitional rehabilitation for 2 months |  |  |  |
|  |  |  |  |  |
| H/O Measles/Whooping Cough | 3-6 months | < 3 months |  |  |  |
| HIV |  | Yes |  |  |  |
| Immunocompromised^c^ | Yes |  |  | Not Improved |  |
| Clinical Manifestation^d^ |  | Suggestive |  | Strongly Suggestive |  |
| Radio-diagnostic Imaging^e^ | Non-specific | Suggestive | Strongly Suggestive |  |  |
| Tuberculin skin test | 5-10 mm |  | > 10 mm |  |  |
| GeneXpert MTB/RIF |  |  |  |  | Positive |
| Micrscope examination of tissue (Histology) | Non-specific |  |  |  | Positive |

^a^Close Contact: History of cough for more than 2 weeks among the household member of the child

^b^PEM/SAM(Protein Energy Malnurtition/Severe Acute Malnurtition: Use WHO recommended Z scoring chart & Not responding to Nurtitional rehabilitation for 2 months

^c^Immunocompromised status: Malignancies like leukamia or lymphoma etc Immunodeficiency diseases like agammaglobunemiaetc, Chemotheraphy/Immunosuppressive therapy such as steriods for more than 2 weeks

^d^Clinical Manifestation: Suggestive of TB: Pulmonary Findings (unilateral wheeze, dullness, weight loss, Hepato-splenomegaly, Lymphadenopathy, ascites etc. Strongly suggestive of TB:Matted Lymph nodes, abdominal mass or doughy abdomen, sinus formation, gibbus formation, chronic mono arthitis, meningeal findings (bulging fonatanel, irritability, choroid tubercule, papilloedema).

^e^Radio-diagnostic/imaging studies includes Chest X-ray, CT Chest/MRI, non-specific signs: ill-defined opacity or patchy infilitrates on chest X-ray, marked broncho-vascular marking, signs suggestive of TB: consolidation not responding to anti-biotic therapy, para-trachael, or mediastinal lymphadenopathy, miliary mottling, cavitation.

**MODIFIED GHIDEY AND HABTE**

| 1. History of contact with a tuberculous adult |
| --- |
| 2. Suggestive symptom complex of TBC |
| 3. Radiological findings compactible with TBC |
| 4. 2 TU PPD reaction positive |
| ≥ 10 mm induration in a non-BCG vaccinated patient |
| ≥15 mm induration in a BCG vaccinated patient |
| 5. Bacteriological or histological proof |
|  |
| **Diagnosis** |
| The presence of 2 or more criteria was required for diagnosis |

**GUNASEKERA 2021 et al ALGORITHM**


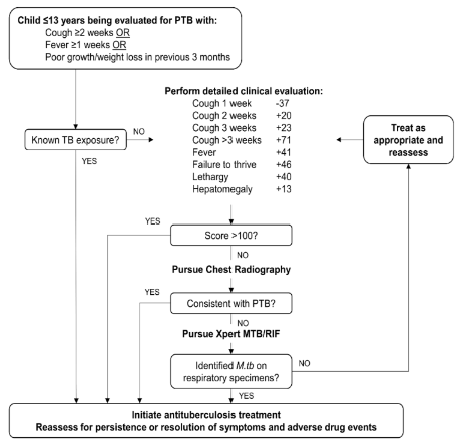


**GUNASEKERA 2023 et al ALGORITHM WITHOUT CXR**


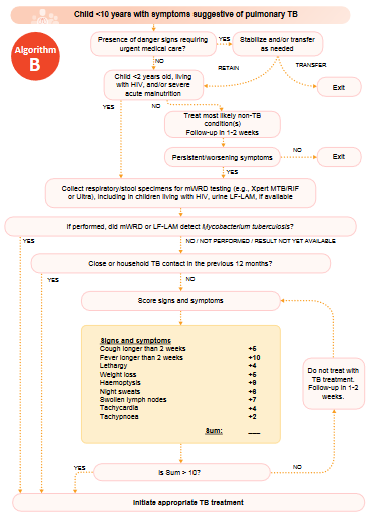


**MARCY et al’s ALGORITHM**


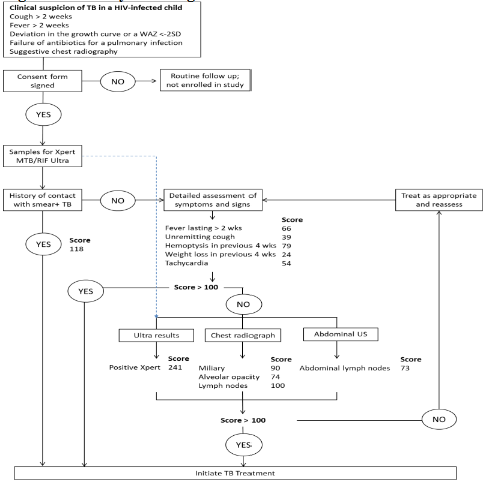


**INTERNATIONAL UNION AGAINST TB AND LUNG DISEASE ALGORITHM**


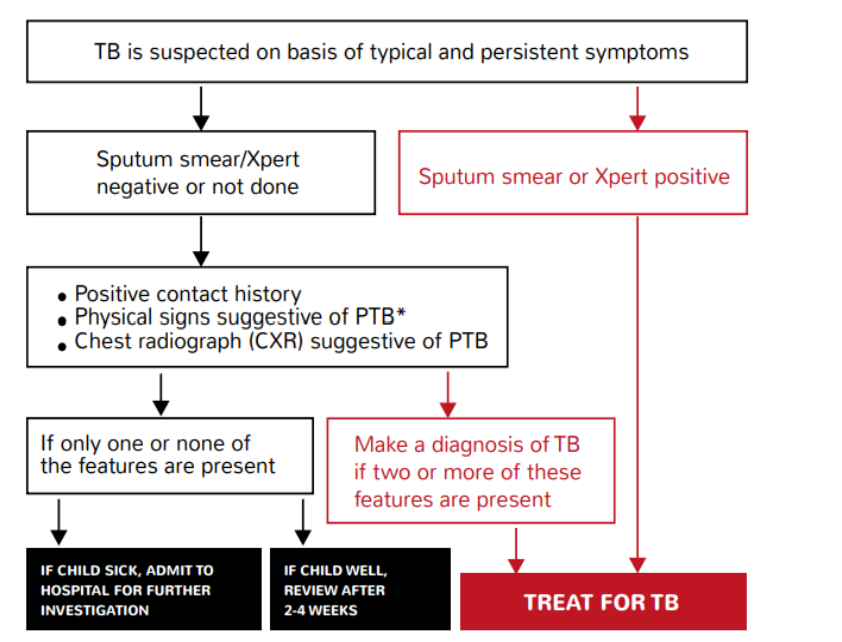

Supplement: ofad624_Supplementary_Data [file ofad624_supplementary_data.doc]
